# Repurposing the Sphingosine-1-Phosphate Receptor Modulator Etrasimod as an Antibacterial Agent Against Gram-Positive Bacteria

**DOI:** 10.3389/fmicb.2022.926170

**Published:** 2022-06-06

**Authors:** Matej Zore, Shella Gilbert-Girard, Paola San-Martin-Galindo, Inés Reigada, Leena Hanski, Kirsi Savijoki, Adyary Fallarero, Jari Yli-Kauhaluoma, Jayendra Z. Patel

**Affiliations:** ^1^Drug Research Program, Division of Pharmaceutical Chemistry and Technology, Faculty of Pharmacy, University of Helsinki, Helsinki, Finland; ^2^Drug Research Program, Division of Pharmaceutical Biosciences, Faculty of Pharmacy, University of Helsinki, Helsinki, Finland

**Keywords:** etrasimod, sphingosine-1-phosphate receptor modulators, repurposing, antimicrobials, biofilms, *Staphylococcus aureus*, Gram-positive bacteria

## Abstract

New classes of antibiotics are urgently needed in the fight against multidrug-resistant bacteria. Drug repurposing has emerged as an alternative approach to accelerate antimicrobial research and development. In this study, we screened a library of sphingosine-1-phosphate receptor (S1PR) modulators against *Staphylococcus aureus* and identified five active compounds. Among them, etrasimod (APD334), an investigational drug for the treatment of ulcerative colitis, displayed the best inhibitory activity against *S. aureus* when growing as free-floating planktonic cells and within biofilms. In follow-up studies, etrasimod showed bactericidal activity and drastic reduction of viable bacteria within 1 h of exposure. It also displayed a potent activity against other Gram-positive bacteria, including penicillin- and methicillin-resistant *S. aureus* strains, *S. epidermidis*, and *Enterococcus faecalis*, with a minimum inhibitory concentration (MIC) ranging from 5 to 10 μM (2.3–4.6 μg/mL). However, no inhibition of viability was observed against Gram-negative bacteria *Acinetobacter baumannii*, *Escherichia coli*, and *Pseudomonas aeruginosa*, showing that etrasimod preferably acts against Gram-positive bacteria. On the other hand, etrasimod was shown to inhibit quorum sensing (QS) signaling in *Chromobacterium violaceum*, suggesting that it may block the biofilm formation by targeting QS in certain Gram-negative bacteria. Furthermore, etrasimod displayed a synergistic effect with gentamicin against *S. aureus*, thus showing potential to be used in antibiotic combination therapy. Finally, no *in vitro* toxicity toward mammalian cells was observed. In conclusion, our study reports for the first time the potential of etrasimod as a repurposed antibacterial compound against Gram-positive bacteria.

## Introduction

The emergence of multidrug-resistant (MDR) bacteria is one of the major threats for public health. Alarmingly, resistance has been observed against all currently used antibiotics, including last-resort antimicrobials such as daptomycin, vancomycin, and linezolid, which are commonly used in life-threatening, multidrug-resistant infections caused by Gram-positive bacteria (Long and Vester, 2012; Ventola, 2015; Miller et al., 2016; De Oliveira et al., 2020; Karaman et al., 2020). Moreover, bacteria can attach to different surfaces and form a biofilm, a multicellular community of microorganisms protected by a self-produced extracellular matrix (Hall-Stoodley et al., 2004; Kumar et al., 2017). Bacterial biofilms are highly tolerant to host immune system and can be up to 1,000 times less sensitive to antibiotic treatment than bacteria in planktonic/single cell state (Kumar et al., 2017). Therefore, it is necessary to find agents that are not only active against planktonic cells, but also able to act on biofilms, either by preventing their formation or by disrupting them (Hansa et al., 2021; Provenzani et al., 2021). However, conventional discovery of antibiotics is a long and expensive process, with a low success rate, and has not been able to cope with the emergence of antibiotic resistance and tolerance (Payne et al., 2007; Ribeiro da Cunha et al., 2019).

Over the past decades, drug repurposing has received increased attention as an attractive strategy for more efficient drug discovery, including antimicrobials (Farha and Brown, 2019). Drug repurposing is a process of finding new therapeutic uses for existing drugs, and it offers several advantages over the conventional drug discovery process. Such advantages are mainly attributed to the fact that approved drugs have already been extensively studied and have known toxicity and pharmacokinetic profiles (Boyd et al., 2021). Moreover, repurposing a drug can reduce costs and risks associated with antimicrobial research and accelerate approval timelines (Farha and Brown, 2019; Boyd et al., 2021).

In a previous study, we found that fingolimod, an FDA-approved drug for the treatment of relapsing-remitting multiple sclerosis, has antibacterial and anti-biofilm activity against *Staphylococcus aureus* (Gilbert-Girard et al., 2020b). Medicinal chemistry efforts by our group provided further insights on structure-activity relationships and yielded several fingolimod derivatives with more potent antibacterial activity (Zore et al., 2021). Fingolimod is a structural analogue of sphingosine, a sphingoid base naturally found in mammalian cells, which has a well reported antimicrobial activity. Sphingosine and other sphingolipids have been investigated against various bacterial species and have been found active against *S. aureus*, *Streptococcus* spp., *Pseudomonas aeruginosa*, *Escherichia coli*, among others (Fischer et al., 2012; Cukkemane et al., 2015; Tavakoli Tabazavareh et al., 2016; Becam et al., 2017). While the antibacterial mechanism of action (MoA) of sphingosine is not yet fully explained, it has been recently reported that sphingosine targets bacterial membrane through binding of the protonated amino group of sphingosine with the negatively charged membrane protein cardiolipin, causing a rapid permeabilization of the bacterial membrane (Tavakoli Tabazavareh et al., 2016; Verhaegh et al., 2020). Since fingolimod contains the same amino-diol functional group, it is likely that fingolimod employs a similar MoA against bacteria as sphingosine. Fingolimod belongs to a class of drugs known as sphingosine-1-phosphate receptor (S1PR) modulators, which are mostly investigated for the treatment of immune-mediated and inflammatory diseases such as multiple sclerosis, Crohn’s disease, and ulcerative colitis (Dyckman, 2017; Marciniak et al., 2018). Since the approval of fingolimod, several other S1PR modulators have been developed, with the aim of improving stability, bioavailability, and efficiency of these modulators (Marciniak et al., 2018; McGinley and Cohen, 2021). Based on our previous findings, we hypothesized that other S1PR modulators that interact with the same receptor could also have a similar antibacterial effect as sphingosine and fingolimod. Thus, we set out to explore the potential of other S1PR modulators as repurposed antibacterial compounds.

Here, we investigated a library of thirteen S1PR modulators and screened it against planktonic cells and biofilms of *S. aureus*, which led us to the identification of etrasimod as the most promising compound. Etrasimod is an immuno-modulating drug candidate developed by Arena Pharmaceuticals, Inc., which has been acquired by Pfizer Inc. in early 2022, and it has mainly been investigated for the treatment of ulcerative colitis (Sandborn et al., 2020; Vermeire et al., 2021). In follow-up studies, we investigated its bactericidal activity against *S. aureus* via time-kill kinetics, as well as its potential to induce bacterial resistance. Then, we used the checkerboard assay to explore synergistic effects of etrasimod with different classes of antibiotics, and thus the possibility of using it in an antibiotic combination therapy. The antibacterial activity of etrasimod against various Gram-positive and Gram-negative bacteria was also investigated. In the case of Gram-negative bacteria, we further studied quorum sensing (QS) inhibitory activity against *Chromobacterium violaceum*, a Gram-negative bacterium commonly used as a QS-bioreporter. Finally, cytotoxicity studies were performed to evaluate toxicity against mammalian cells. Overall, our study highlights the potential of S1PR modulator etrasimod as an antibacterial and anti-biofilm compound.

## Materials and Methods

### Bacterial Strains and Culture Conditions

*S. aureus* (ATCC 25923, Newman and ATCC 43300), *S. epidermidis* RP62A (ATCC 35984), *P. aeruginosa* (ATCC 15442 and PAO1), and *E. coli* ATCC BAA1161 were provided by the Faculty of Pharmacy, University of Helsinki, Finland. *Enterococcus faecalis* ATCC 29212 was bought from Microbiologics Inc. (St. Cloud, MN, United States). *Acinetobacter baumannii* NCTC 13423 and *C. violaceum* NCTC 13278 (Tn5-mutant CV026) were bought from the National Collection of Type Culture, NCTC (Salisbury, United Kingdom). *C. violaceum* ATCC 31532 was bought from the American Type Culture Collection, ATCC (Wesel, Germany). *S. aureus* P2 and *S. epidermidis* P55 were isolated from orthopedic prostheses at the Hospital Fundación Jiménez Díaz (Madrid, Spain) and were kindly given by Ramón Pérez-Tanoria. All bacterial strains were stored as cryogenic stocks (−80 °C). Prior to each experiment, every strain (except for *P. aeruginosa*, *E. coli*, and *C. violaceum*) was first grown overnight at 37 °C on a tryptic soy agar (TSA; Neogen, Lansing, MI, United States) plate. Afterward, the colonies were dispersed in 5 mL of tryptic soy broth (TSB; Neogen, Lansing, MI, United States) and incubated at 37 °C with shaking (220 rpm), until the culture reached a concentration of approximately 1 × 10^8^ colony-forming unit (CFU)/mL. The bacterial concentration was determined by measuring the optical density at 595 nm using Multiskan GO spectrophotometer (Thermo Fisher Scientific, Waltham, MA, United States), followed by 10-fold serial dilutions and plate counting. The cultures were diluted to approximately 1 × 10^6^ CFU/mL before starting the experiment. The same was done for *P. aeruginosa* and *E. coli* using Lennox broth (LB) and LB-agar (LBA). *C. violaceum* was grown overnight on LBA at 27 °C and the colonies were used to start the experiment directly.

### Compounds

For the screening, all commercially available S1PR modulators were purchased from Cayman Chemical (Ann Arbor, MI, United States), except for KRP-203 and fingolimod, which were purchased from Carbosynth (Compton, United Kingdom). According to the manufacturer’s claims, all compounds have a purity of at least 95%. Prior to screening, compounds were diluted in DMSO (VWR, Radnor, PA, United States). For the follow-up studies, etrasimod was purchased from Carbosynth (Compton, United Kingdom). Antibiotics for resistance development and checkerboard assay [dicloxacillin (D9016), vancomycin (861987), ciprofloxacin (17850), rifampicin (R3501), and gentamicin (48760)] were purchased from Sigma-Aldrich (St. Louis, MO, United States).

### Antibacterial Activity Evaluation

The antibacterial activity was evaluated under two modes of exposure (pre- and post-exposure) (Fallarero et al., 2013). In pre-exposure, the compounds were plated in 96-well plates (Nunclon D surface, 167008, Thermo Fisher Scientific, Waltham, MA, United States) at concentrations ranging from 2.5 to 50 μM (final concentration of 1% DMSO in the wells). Then, of bacterial culture was added and the plates were incubated under aerobic conditions for 18 h at 37 °C with shaking (220 rpm). Planktonic and biofilm growth was assessed using two different measurements for each: optical density (turbidity) and resazurin reduction (viability) on planktonic cells, and resazurin reduction and crystal violet staining (total biomass) on biofilms, as described in the following sections. Minimum inhibitory concentration (MIC) is defined here as the lowest concentration that prevented bacterial growth, resulting in over 90% inhibition of turbidity and viability of planktonic cells. In post-exposure, bacteria were first grown for 18 h with no compound under the same incubation conditions (37 °C, 220 rpm). Afterward, the media was changed, and various concentrations of compounds (50–200 μM) were added to the preformed biofilms. The plates were incubated for an additional 24 h at 37 °C with shaking (220 rpm) before proceeding with the staining assays.

### Resazurin Staining

Resazurin staining was performed according to previously optimized protocols with a few modifications (Skogman et al., 2012; Gilbert-Girard et al., 2020a). Briefly, the planktonic solution was transferred into a clean 96-well plate and the OD at 595 nm was measured using a Multiskan GO spectrophotometer (Thermo Fisher Scientific, Waltham, MA, United States). Afterward, 10 μL of resazurin (400 μM; R7017, Sigma-Aldrich, St. Louis, MO, United States) diluted in phosphate-buffered saline (PBS) was added to the wells. The plate was incubated in the dark with shaking (220 rpm) for about 3–10 min at room temperature (RT) with *S. aureus*, *S. epidermidis*, and *A. baumannii*, for 20 min at 37 °C with *P. aeruginosa* and *E. coli*, or for 60 min at 37 °C with *E. faecalis*. Fluorescence was measured at λ_*ex*_ = 560 nm and λ_*em*_ = 590 nm using the top optics of the Varioskan LUX Multimode microplate reader (Thermo Fisher Scientific, Waltham, MA, United States). The planktonic plate was then discarded. The original plate containing the biofilms was washed once with PBS, and 200 μL of a 20 μM resazurin solution in PBS was added to the wells. The plate was incubated in the dark at 37 °C with shaking (220 rpm) for 30 min with *S. aureus* and *S. epidermidis*, 60 min with *A. baumannii*, and 90 min with *E. faecalis, P. aeruginosa, and E. coli*. Fluorescence was measured as described for the planktonic solution. The biofilm biomass was next stained with crystal violet.

### Crystal Violet Staining

The resazurin solution was removed and the biofilms were fixed with 100% EtOH for 15 min at RT. Then, the EtOH was removed, and biofilms were left to dry completely at RT. Biofilm biomass was stained with 0.02% crystal violet solution (prepared from 1% commercial solution; V5265, Sigma-Aldrich, St. Louis, MO, United States), incubated for 5 min at RT, washed twice with MQ-water and air-dried for 10 min. The bound dye was dissolved in 100% EtOH for 1 h, and absorbance was measured at 595 nm with a Multiskan GO spectrophotometer (Thermo Fisher Scientific, Waltham, MA, United States).

### Viable Cell Count in Biofilms

*S. aureus* ATCC 25923 biofilms were grown in 96-well plates for 18 h (37 °C, 220 rpm). Afterward, the media was changed, and the biofilms were exposed to etrasimod at the concentration of 25–200 μM for 24 h (37 °C, 220 rpm). Untreated bacteria were used as negative control. The planktonic solution was removed, and biofilms were washed once with PBS before being scraped with a pipette tip in 100 μL of PBS. Then, the bacteria were serially diluted in PBS and plated on TSA. Colonies were counted after an overnight incubation at 37 °C.

### Minimum Bactericidal Concentration and Biofilm Preventing Concentration

*S. aureus* ATCC 25923 was exposed to various concentrations of etrasimod (2.5–50 μM) in a 96-well plate in similar conditions to the pre-exposure assay, as described above for antibacterial activity evaluation. Untreated bacteria and bacteria exposed only to the solvent (1% DMSO) were used as controls. To determine MBC, in wells without visible growth (from the MIC and higher), aliquot of planktonic solution was serially diluted in TSB and plated on TSA. To determine BPC, the remaining planktonic solution was removed, and wells were washed once with PBS. The bottom of the wells was scraped in 100 μL of PBS with a pipette tip, serially diluted in PBS and plated on TSA. Colonies were counted after an overnight incubation at 37 °C. The MBC is defined as the lowest compound concentration killing ≥99.9% of planktonic bacteria, whereas BPC is the lowest compound concentration preventing the adherence and survival of ≥99.9% of bacterial cells on the surface of the wells.

### Time-Kill Kinetic Assay

*S. aureus* ATCC 25923 was grown overnight, and then diluted to a concentration of 1 × 10^6^ CFU/mL. In a 15-mL Falcon tube, 4 mL of bacterial culture was added, followed by addition of etrasimod at different concentrations in relation to its MIC (5–10–20–40–160 μM). Bacteria exposed to the solvent alone (1% DMSO) were used as a growth control. The tubes were incubated at 37 °C with shaking (220 rpm), and a 200 μL sample was collected from each tube at various time points (0.25, 0.5, 1, 2, 3, 4, 6, 8, 12, and 24 h). The samples were centrifuged (10,000 × g, 4 °C, 5 min) and the supernatant was removed. Bacteria pellets were dispersed in 200 μL of PBS, transferred to a 96-well plate, and OD_595_ was measured using Multiskan GO spectrophotometer (Thermo Fisher Scientific, Waltham, MA, United States). Bacteria was then serially diluted in PBS, plated on TSA, and colonies were counted after an overnight incubation at 37 °C.

### Resistance Development Assay

*S. aureus* ATCC 25923 was grown overnight, and then diluted to a concentration of 1 × 10^6^ CFU/mL. Etrasimod and antibiotic controls (dicloxacillin and vancomycin) were plated in a 96-well plate at MIC and 0.5 × MIC, followed by addition of 200 μL of bacterial culture. The plate was incubated for 24 h (37 °C, 220 rpm), and then the bacterial growth was visually assessed. An aliquot of 10 μL was transferred from the well with the highest concentration of each compound with visible growth (either 0.5 × MIC or the MIC, if resistance was developed) into two wells containing 190 μL of fresh TSB. Compounds were added at the same concentrations as previously or 2-fold higher if the MIC had increased. The procedure was repeated sequentially in the same manner until 20 days were reached.

### Synergy Testing

The synergistic effect of etrasimod with antibiotics (vancomycin, dicloxacillin, ciprofloxacin, rifampicin, and gentamicin) was determined by the checkerboard assay. Combinations of etrasimod and antibiotics were prepared in a 96-well plate, starting with a concentration 2-fold higher than their MIC, and then serially diluted in a 2-fold manner. Suspension of *S. aureus* ATCC 25923 at 1 × 10^6^ CFU/mL in TSB was added, and the plate was incubated for 18 h (37 °C, 220 rpm). Planktonic solution was transferred to a 96-well plate and OD_595_ was measured with Multiskan GO spectrophotometer (Thermo Fisher Scientific, Waltham, MA, United States). Then, 10 μL of resazurin (400 μM in PBS) was added to the wells, the plate was incubated in the dark (5 min, RT, 220 rpm) and fluorescence was measured at λ_*ex*_ = 560 nm and λ_*em*_ = 590 nm using a Varioskan LUX Multimode microplate reader (Thermo Fisher Scientific, Waltham, MA, United States). MIC was determined as the lowest concentration of etrasimod/antibiotic combination that caused over 90% inhibition of turbidity and viability of planktonic cells compared to untreated bacteria. The combinatory effect of each etrasimod/antibiotic combination was determined by calculating the fractional inhibitory concentration index (FICI), according to the following equation:


$$\text{FICI}=\,\frac{\mathrm{MIC}\,\left(\mathrm{etrasimod}\,\mathrm{in}\,\mathrm{combination}\right)}{\mathrm{MIC}\,\left(\mathrm{etrasimod}\,\mathrm{alone}\right)}+\frac{\mathrm{MIC}\,\left(\mathrm{antibiotic}\,\mathrm{in}\,\mathrm{combination}\right)}{\mathrm{MIC}\,\left(\mathrm{antibiotic}\,\mathrm{alone}\right)}$$

Fractional inhibitory concentration index was interpreted as: ≤0.5 = synergy, >0.5–4 = no interaction, and ≥4 antagonism (Odds, 2003).

### Quorum Sensing Inhibition Assay

The QS inhibitory activity of etrasimod was determined as reported previously (Skogman et al., 2016; Beus et al., 2020). Briefly, *C. violaceum* ATCC 31532 and the violacein-negative, mini-Tn5 mutant of *C. violaceum* CV026 (NCTC 13278), were grown overnight on LBA at 27 °C. For CV026, the LBA was supplemented with kanamycin at 100 μg/mL. Colonies were dispersed in PDYT (0.5% peptone, 0.3% D-glucose, 0.25% yeast extract, 0.05% L-tryptophan, w/v) to reach an OD_600_ of 0.02. The CV026 culture was supplemented with 0.5 μM C6-HSL (*N*-hexanoyl-L-homoserine lactone; 10007896, Cayman Chemical, Ann Arbor, MI, United States) to induce the QS-moderated synthesis of violacein. For each strain, compounds were plated in two identical 96-well plates, followed by addition of 200 μL of bacterial culture per well. In each plate, untreated cells were used as negative controls, azithromycin (PZ0007, Sigma-Aldrich, St. Louis, MO, United States) was used as a positive control for bactericidal activity, and quercetin (Q4951, Sigma-Aldrich, St. Louis, MO, United States) as a positive control for QS inhibition. The plates were incubated for 22 h at 27 °C with shaking (200 rpm). The first 96-well plate was centrifuged (4,000 rpm, for 15 min, 20 °C) to collect the synthesized and insoluble violacein. Supernatants were removed, violacein was dissolved in 100 μL per well of 96% (v/v) EtOH and was separated from cells by centrifugation (4,000 rpm, for 15 min, 20 °C). The supernatant containing violacein was then transferred to a new 96-well plate, and the absorbance was measured at 595 nm using a Multiskan Sky Microplate spectrophotometer (Thermo Fisher Scientific, Waltham, MA, United States). In the second replica plate, the viability of the cells was measured by adding 10 μL of resazurin (400 μM in PBS) in each well and incubating the plate at 27 °C (220 rpm) in the dark for 30 min. After centrifugation of the plate to separate the cells from the solution, 100 μL of each well were transferred into a clean 96-well plate and the fluorescence was recorded at λ_*ex*_ = 560 nm and λ_*em*_ = 590 nm using a Varioskan LUX Multimode microplate reader (Thermo Fisher Scientific, Waltham, MA, United States).

### Human Cell Lines and Maintenance

The human promyelocytic leukemia cell line, HL-60 (ATCC CCL-240), was grown and maintained in 72 cm^2^-culture flasks suspended in Roswell Park Memorial Institute (RPMI) 1640 Medium (R8758, Sigma-Aldrich, St. Louis, MO, United States) supplemented with 1% (v/v) penicillin/streptomycin (Sigma-Aldrich, St. Louis, MO, United States) and 20% (v/v) heat-inactivated fetal bovine serum (FBS; Sigma-Aldrich, St. Louis, MO, United States). The cell density was maintained within 10^5^–10^6^ cells/mL. For the cytotoxicity assay, the cells were differentiated into polymorphonuclear-like cells. To do so, the cells were incubated for 6 days in the maintenance medium with *N,N*-dimethylformamide (DMF; Sigma-Aldrich, St. Louis, MO, United States) at a concentration of 100 mM (Reigada et al., 2020). The human lung adenocarcinoma epithelial cells A549 (CCL-185, ATCC, Wesel, Germany) were grown in Dulbecco’s Modified Eagle Medium (DMEM; Lonza, Basel, Switzerland) supplemented with 10% FBS (Sigma-Aldrich, St. Louis, MO, United States), 2 mM L-glutamine (Sigma-Aldrich, St. Louis, MO, United States) and 20 μg/mL of gentamicin (Lonza, Basel, Switzerland). Both cell lines were kept at 37 °C in 5% CO_2_ in a humidified incubator (Heracell™ 240i CO_2_ Incubator, Thermo Fisher Scientific, Waltham, MA, United States).

### Cytotoxicity Studies

Both HL-60 cells and A549 cells were seeded on a 96-well plate at a concentration of 1 × 10^4^ cells/well in a total volume of 200 μL/well. After 24-h incubation the media of the A549 was changed and the compound was added to both cell lines at final concentrations of 1–100 μM, being the maximum DMSO concentration used 0.25%. The negative control consisted of cells treated with the highest concentration of DMSO and the positive control of cells treated with 100 μM usnic acid (Sigma-Aldrich, St. Louis, MO, United States). Cells were further incubated for 24 h, after which the media of the adherent cells was removed, and the cells washed once with PBS. Then, 200 μL of a 20 μM resazurin solution in PBS was added per well. In the case of the HL-60 cells, 10 μL of a resazurin solution (400 μM) was added per well with no change of media. Cells were incubated for 2 h, after which the fluorescence was measured at λ_*ex*_ = 560 nm and λ_*em*_ = 590 nm using a Varioskan LUX Multimode microplate reader (Thermo Fisher Scientific, Waltham, MA, United States).

### Statistical Analysis

The assay performance was monitored by calculating the screening window coefficient (Z′) (Zhang et al., 1999). Statistical significance of the results was determined using a one-way ANOVA with the Welch correction and a *post hoc* Dunnett’s test. Significance was indicated as follows: *p < 0.05, ^**^p < 0.01, and ^***^p < 0.001. Results were processed with IBM SPSS Statistics 28.0.0.0.

## Results and Discussion

### Antibacterial Screening of Sphingosine-1-Phosphate Receptor Modulators

We first assembled a library of thirteen S1PR modulators. The main criteria for selecting the compounds were structural diversity and commercial availability. The library consisted of three FDA-approved drugs (siponimod, ozanimod, and ponesimod), while the other compounds were investigational drugs, with many of them having already entered clinical trials (Dyckman, 2017; Marciniak et al., 2018; McGinley and Cohen, 2021; Roy et al., 2021). The initial screening of the S1PR modulators was carried out at the concentration of 50 μM against planktonic cells and biofilms of *S. aureus* ATCC 25923, using fingolimod as a reference. Compounds that inhibited both planktonic growth (turbidity and viability) and biofilm formation (viability and biomass) by at least 80% were considered active. From the initial screening, we identified five active compounds: amiselimod, etrasimod, KRP-203, AUY954, and GSK 2018682 (Table 1). All chemical structures and inhibition results of each compound are available in Supplementary Table 1.

**TABLE 1:** Antibacterial activity of the five active sphingosine-1-phosphate receptor (S1PR) modulators against *Staphylococcus aureus* ATCC 25923.

Compound	Structure	MIC μM (μg/mL)*^a^*
	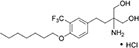	
Amiselimod		15 (5.7)
	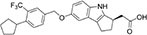	
Etrasimod		5–10 (2.3–4.6)
	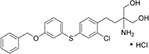	
KRP-203		25 (11.1)
	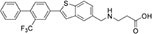	
AUY954		50 (22.8)
	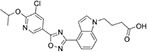	
GSK2018682		15 (6.6)

*^a^Results are the average of three biological repetitions, each with two technical replicates per concentration.*

We next searched the literature to assess the potential of the active compounds as repurposed antibacterial agents. According to our literature search, none of the five active compounds had been previously studied for their antimicrobial activity. Therapeutic potential of amiselimod, etrasimod, and KRP-203 has been investigated for various autoimmune diseases, including multiple sclerosis, Crohn’s disease, ulcerative colitis, psoriasis, and inflammatory bowel disease (Dyckman, 2017; McGinley and Cohen, 2021; Roy et al., 2021). Compared to fingolimod, amiselimod showed improved cardiac safety profile and reduced fingolimod-associated bradycardia (Sugahara et al., 2017). Amiselimod and KRP-203 are structural analogs of fingolimod, thus it is not surprising that they were identified as active compounds in our study. Since they both contain amino-diol functional group, it is possible that they employ a similar mechanism of action against bacteria as fingolimod. Etrasimod (APD334), developed by Arena Pharmaceuticals, Inc., has been mainly investigated as a drug candidate for the treatment of ulcerative colitis, and in clinical trials, it showed good efficacy and favorable safety profile (Sandborn et al., 2020; Vermeire et al., 2021). GSK2018682 has been developed for the treatment of relapsing-remitting multiple sclerosis, and phase 1 trial was completed in 2011 (Xu et al., 2014), however, its development has been discontinued since. GSK2018682 and etrasimod contain indole and cyclopenta[*b*]indole core, respectively, which are considered as promising scaffolds with a broad range of biological activities, including antibacterial effects (Samosorn et al., 2006; Lepri et al., 2016; Amuthavalli et al., 2020; Chen et al., 2021). Finally, AUY954 was shown to prevent transplant rejection in mice (Pan et al., 2006), but to the best of our knowledge, it has not yet reached clinical trials.

We further determined the minimum inhibitory concentration (MIC) of the five active compounds from the initial screening, by testing them in pre-exposure at 2.5–25 μM against *S. aureus* ATCC 25923. Table 1 shows MIC values obtained for five active compounds. All inhibition results are available in Supplementary Table 2. Fingolimod was previously reported to have an MIC of 15 μM, with some activity on biofilms as well (Gilbert-Girard et al., 2020b). Among active S1PR modulators, etrasimod showed the highest activity, with the MIC ranging between 5 and 10 μM (depending on the replicate), even exceeding the activity previously obtained by fingolimod. Amiselimod, and KRP-203, both fingolimod derivatives, also had a good activity against *S. aureus*, with an MIC of 15 and 25 μM, respectively. GSK2018682 had the same MIC as fingolimod, while AUY954 displayed a much lower activity, with an MIC of 50 μM. Since etrasimod displayed the best inhibitory activity against *S. aureus*, it was chosen for follow-up *in vitro* studies.

### Anti-biofilm Activities of Active Sphingosine-1-Phosphate Receptor Modulators

**FIGURE 1:**
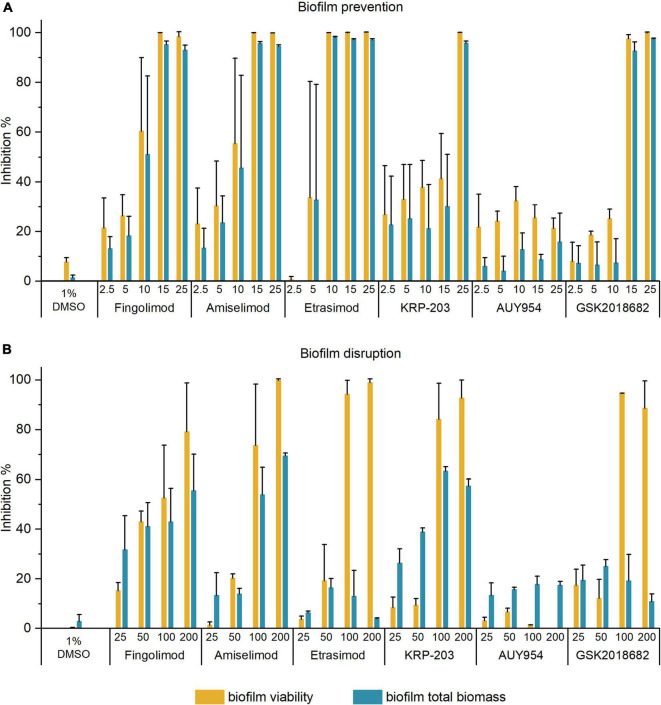
Inhibition of biofilm viability and biofilm biomass of *Staphylococcus aureus* biofilms in panel **(A)** pre-exposure; and **(B)** post-exposure assay by different concentrations of active S1PR modulators. The results are expressed as the average inhibition percentage ± SD of two or three biological repetitions, each with two or three technical replicates.

The five active S1PR modulators were further tested for their anti-biofilm activity against *S. aureus* biofilms in both pre-exposure (prevention of biofilm formation) and post-exposure (disruption of pre-formed biofilms) by measuring biofilm viability and total biomass. In the pre-exposure assay (Figure 1A), all five compounds inhibited biofilm formation at their respective MICs, resulting in over 90% inhibition of biofilm viability and total biofilm biomass. All inhibition results are available in Supplementary Table 2.

In the post-exposure assay, we investigated the ability of compounds to disrupt 18 h-old pre-formed biofilms. Figure 1B shows the inhibition of biofilm viability and total biomass of *S. aureus* biofilms by the active S1PR modulators at 25–200 μM. All inhibition results are available in Supplementary Table 3. Fingolimod was again used as a reference compound and benchmarked against the previous reports (Gilbert-Girard et al., 2020b). Etrasimod and GSK2018682 both inhibited biofilm viability of pre-formed biofilms by at least 90% at 100 μM, even exceeding the activity observed with fingolimod. On the other hand, they both failed to reduce the total biomass (inhibition between 20 and 30% up to 200 μM). Fingolimod derivatives amiselimod and KRP-203 reduced biofilm viability by at least 70 and 80%, respectively, at 100 μM, also exceeding the activity observed with fingolimod. In addition, they reduced the total biofilm biomass of established biofilms by 50–60% at 100 μM. Considering that biofilms are much more tolerant to chemical agents, such inhibition is not negligible. On the other hand, AUY954 did not affect pre-formed biofilms, as it inhibited biofilm viability and total biomass by less than 20%, despite having some antibacterial activity against planktonic cells in pre-exposure.

**FIGURE 2:**
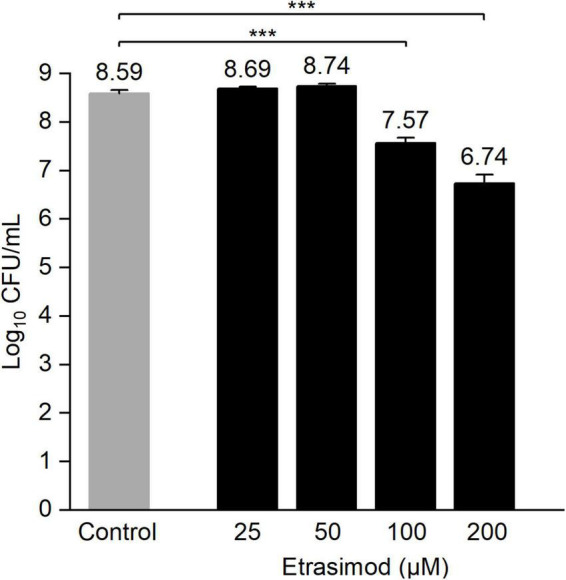
Log_10_ of the colony-forming unit (CFU) count of *Staphylococcus aureus* biofilms exposed to different concentrations of etrasimod for 24 h in post-exposure. Untreated biofilms were used as controls. Results are the average of three biological repetitions, each with two biofilm replicates per concentration (***p < 0.001).

As etrasimod displayed the highest biofilm viability inhibitory activity, we decided to further investigate its activity against biofilms. The inhibition percentages obtained in Figure 1B differed greatly between biofilm viability and total biomass. Since resazurin staining depends on metabolically active cells, dormant cells with reduced metabolic activity will most likely not be detected using this method. On the other hand, crystal violet stains both extracellular matrix and live/dead cells but does not give information about viability of the cells in the biofilm. Thus, to confirm if the reduced biofilm viability observed with resazurin (Figure 1B) corresponded to a reduced number of viable cells within the pre-formed biofilm, we performed a viable cell count after a 24-h treatment. Figure 2 shows the concentration-dependent reduction of viable cells in pre-formed biofilm. Etrasimod caused a 1-log reduction at 100 μM and close to a 2-log reduction at 200 μM, which, respectively, represent 90 and 99% reduction of the number of cells within the biofilm. This confirms that etrasimod reduced the number of live cells in a pre-formed biofilm.

### Antibacterial Activity of Etrasimod Against Planktonic Bacteria

Etrasimod displayed the best antibacterial activity of all the tested S1PR modulators, thus we selected it for further *in vitro* characterization. To determine if the antibacterial activity of etrasimod was bacteriostatic or bactericidal, we measured the minimum bactericidal concentration (MBC) and biofilm preventing concentration (BPC) by performing CFU counts in the wells where no bacterial growth was visible after pre-exposure treatment. Etrasimod had MBC and BPC values equal to its MIC (10 μM), proving that it had a bactericidal activity against *S. aureus* ATCC 25923 and prevented adhesion and survival of the bacterial cells on the surface of the wells as well.

**FIGURE 3:**
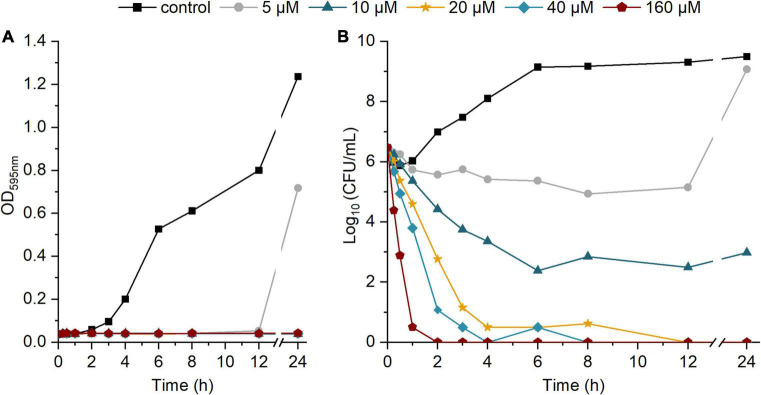
Time-kill kinetic of different concentrations of etrasimod against *Staphylococcus aureus* ATCC 25923 over 24-h incubation: **(A)** OD_595_ of the bacterial culture over time; **(B)** Log_10_ of the CFU/mL in the bacterial culture over time. Results are the average of four biological repetitions, each with one replicate per concentration.

To further investigate the bactericidal activity, we performed a time-kill assay in which *S. aureus* ATCC 25923 was exposed to different concentrations of etrasimod (5–160 μM), for 24 h. The time-kill curve shows a concentration-dependent bacterial death (Figure 3). The curve of the OD over time shows that all tested concentrations inhibited visible growth during the first 12 h of incubation. Only the bacteria exposed to 5 μM showed an increasing OD and CFU value between 12 and 24 h of treatment, thus following the trend of the bacterial control. All concentrations from the 10 μM and above reduced the number of viable cells already during the first hour of exposure and caused at least a 3-log reduction of live cells after 3 h, which represents over 99.9% reduction of the viable cells. At 10 μM, etrasimod caused more than a 6-log reduction after the 24-h incubation. Concentrations from 20 μM and higher reduced number of viable cells already during first 30 min of exposure, killed all bacteria within 12 h, and no re-growth was observed. Altogether, these results show that etrasimod quickly reduces the number of viable bacteria in a culture, suggesting that it may affect the bacterial cell by possibly interfering in the cell wall synthesis or causing a membrane disruption.

### Resistance Development

Bacterial resistance to antibiotics can greatly reduce the useful life of a novel antibiotic and certain compounds have higher potential to induce resistance than others. We therefore evaluated whether *S. aureus* would easily develop resistance against etrasimod when exposed to a sub-inhibitory dose over 20 days. The starting MIC of etrasimod was 10 μM, and we used dicloxacillin (MIC 0.25 μM) and vancomycin (MIC 2.5 μM) as reference antibiotics.

**FIGURE 4:**
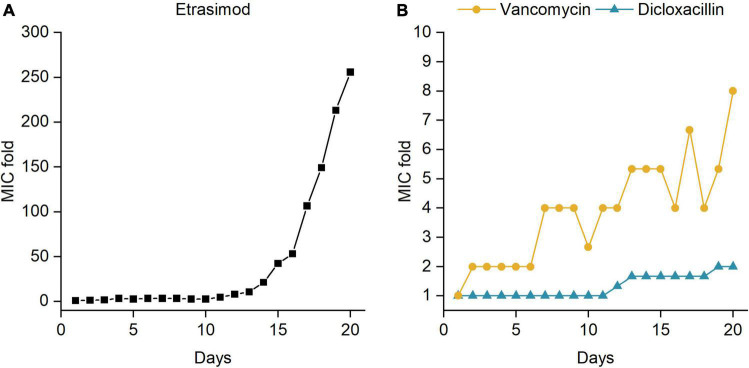
Changes in minimum inhibitory concentration (MIC) over time for *Staphylococcus aureus* ATCC 25923 exposed to sub-inhibitory concentrations of **(A)** etrasimod; and **(B)** vancomycin and dicloxacillin for 20 days. The experiment was repeated with three biological replicates.

Results of the resistance development assay are shown in Figure 4. Dicloxacillin was the best performing antibiotic in this study, since only a 2-fold increase in MIC was observed after 20 days, in contrast to the vancomycin that had an 8-fold increase in MIC. Interestingly, the MIC of etrasimod remained within 3-fold until day 10. After that, the resistance started to develop very quickly, as shown by the rapid increase in MIC. By the end of the experiment, the MIC of etrasimod increased by over 250-fold, suggesting that the *S. aureus* cultures were fully resistant. Moreover, the concentration used was becoming too high to allow full dissolution of the compound. These results suggest that etrasimod could potentially be used for a short-course monotherapy treatment. For longer antibiotic treatments, in order to prevent or minimize the chances of resistance development, it would be more reasonable to use it in a combination with another antibiotic, a possibility we investigated next.

### Combination Effects of Etrasimod With Different Classes of Antibiotics

As mentioned, considering the results of the resistance development assay, it would not be advisable to use etrasimod as a monotherapy for longer therapy schedules. Therefore, we investigated if etrasimod could be used in a combination therapy with conventional antibiotics. We used the checkerboard assay to assess the degree of synergy between different combinations of etrasimod and antibiotics from five different classes. Prior to this assay, we determined the MICs of each antibiotic (Table 2). Combinations of etrasimod and antibiotics were prepared in a 96-well plate, starting with a concentration 2-fold higher than their MIC, followed by three or four 2-fold dilutions. Etrasimod exhibited a synergistic effect with gentamicin, with a fractional inhibitory concentrations index (FICI) of 0.5. The MICs of etrasimod and gentamicin in combination were reduced by 4-fold, when compared to the MICs of the compounds alone. The combination of vancomycin and etrasimod had no interaction (FICI 0.75). However, the MICs of vancomycin and etrasimod were reduced by 2- and 4-fold, respectively, suggesting a potential additive effect (Jenkins and Schuetz, 2012). On the other hand, there was no interaction between etrasimod and dicloxacillin, ciprofloxacin and rifampicin (FICI 2), while none of the combinations resulted in antagonism (FIC > 4.0). This study shows the potential of using etrasimod in a combination therapy, especially with gentamicin, against *S. aureus* infections, and thus reducing the likelihood of developing resistance. Such antibiotic combination therapy could also help overcoming some of the potential limitations associated with etrasimod monotherapy through lowering the required therapeutic doses. Furthermore, since the clinical use of gentamicin is limited due to nephrotoxicity, such combination therapy could potentially decrease the risk of toxicity by reducing the required doses of gentamicin. However, further studies would be needed to confirm these hypotheses.

**TABLE 2:** Minimum inhibitory concentrations of the five antibiotic agents against *Staphylococcus aureus* ATCC 25923, alone and in combination with etrasimod.

Antibiotic	MIC antibiotic μM (μg/mL)		MIC etrasimod μM (μg/mL)		FICI	Interpretation
	Alone	Combination	Alone	Combination		
Vancomycin	2.5 (3.6)	1.25 (1.8)	5 (2.3)	1.25 (0.57)	0.75	No interaction
Dicloxacillin	0.25 (0.1)	0.25 (0.1)	5 (2.3)	5 (2.3)	2	No interaction
Ciprofloxacin	1.25 (0.4)	1.25 (0.4)	5 (2.3)	5 (2.3)	2	No interaction
Rifampicin	0.02 (0.016)	0.02 (0.016)	5 (2.3)	5 (2.3)	2	No interaction
Gentamicin	n.a.*^a^* (2)	n.a. (0.5)	5 (2.3)	1.25 (0.57)	0.5	Synergy

*^a^For gentamicin only mass concentration is reported.*

### Activity of Etrasimod Against Other Staphylococcus aureus Strains and Gram-Positive Species

We tested the antibacterial activity of etrasimod in pre-exposure against additional *S. aureus* strains, as well as *S. epidermidis* and *E. faecalis*. We used the same combination of four measurements as for the initial screening of S1PR modulators (planktonic turbidity and viability, biofilm viability, and total biofilm biomass). All inhibition results are available as Supplementary Table 4. Etrasimod was tested against clinical strains of *S. aureus*, including Newman, which was first isolated in 1952 from a human infection (Duthie and Lorenz, 1952), P2, a penicillin-resistant strain isolated for prosthetic hip implant infection (Esteban et al., 2010), and ATCC 43300, a clinical reference methicillin- and oxacillin-resistant (MRSA) strain. As shown in Table 3, etrasimod exhibited similar and even increased activity with the MIC of 5 μM (2.3 μg/mL) against all clinical *S. aureus* strains compared to *S. aureus* ATCC 25923. Importantly, drug-resistant strains were also susceptible to etrasimod, suggesting different MoA than common antibiotics. Similar activity was observed also against *S. epidermidis*, with an MIC of 10 μM against reference RP62A strain and penicillin-resistant clinical strain P55 (Esteban et al., 2010). Additionally, etrasimod showed high inhibitory activity against *E. faecalis*, with an MIC of 5 μM. Furthermore, at its respective MIC, etrasimod inhibited biofilm formation of all tested strains, resulting in over 90% inhibition of biofilm viability and total biofilm biomass.

**TABLE 3:** Minimum inhibitory concentrations of etrasimod against tested Gram-positive bacterial species.

Etrasimod MIC μM (μg/mL)*^a^*					
*Staphylococcus aureus* Newman	*Staphylococcus aureus* P2*^b^*	*Staphylococcus aureus* ATCC 43300*^c^*	*Staphylococcus epidermidis* RP62A	*Staphylococcus epidermidis* P55*^b^*	*Enterococcus faecalis* ATCC 29212
5 (2.3)	5 (2.3)	5 (2.3)	10 (4.6)	10 (4.6)	5 (2.3)

*^a^Results are average of three biological repetitions, each with two technical replicates per concentration.*

*^b^Penicillin-resistant strain.*

*^c^Methicillin- and oxacillin-resistant strain.*

### Activity of Etrasimod Against Gram-Negative Species

We further investigated the spectrum of antibacterial activity of etrasimod by testing it in pre-exposure against Gram-negative bacteria *A. baumannii* NCTC 13423, *E. coli* ATCC BAA1161 and two strains of *P. aeruginosa* (ATCC 15442 and PAO1). All inhibition results are available in Supplementary Table 5. Etrasimod did not display any noteworthy inhibition of bacterial turbidity and viability, as well as biofilm formation up to concentration of 200 μM against any of these species. As etrasimod is a hydrophobic compound, it is possible that the lipopolysaccharide layer of Gram-negative bacteria prevented it from entering the bacterial cells and exerting its antibacterial effect (Zhang et al., 2013). Therefore, we concluded that etrasimod preferably acts against Gram-positive bacteria.

### Quorum Sensing Inhibitory Activity of Etrasimod

**FIGURE 5:**
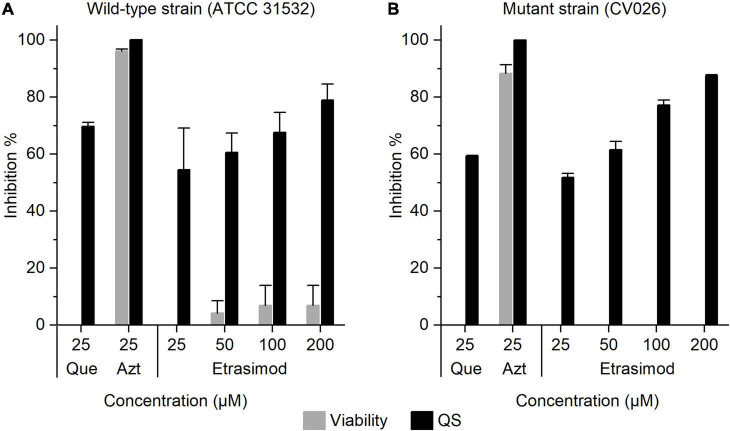
Inhibition of the viability and QS activity of *Chromobacterium violaceum* **(A)** wild-type strain (ATCC 31532); and **(B)** mutant strain CV026 by etrasimod, quercetin (Que, QSI control) and azithromycin (Azt, bactericidal control). The results are expressed as the inhibition percentage ± SD. The experiment was repeated with two biological repetitions, each with two replicates per concentration.

Quorum sensing (QS) is a bacterial communication system coordinating specific community behavior, such as biofilm formation (Waters and Bassler, 2005). Inhibiting QS is an interesting alternative approach to reduce the drug use in infections involving biofilms, as preventing biofilm formation maintains bacteria in a more susceptible planktonic state. The QS inhibitory activity of fingolimod and its derivatives has been previously demonstrated by our group (Gilbert-Girard et al., 2020b; Zore et al., 2021). To investigate whether etrasimod could also act as quorum sensing inhibitor (QSI), we tested it using a previously described *C. violaceum* platform (Skogman et al., 2016). *C. violaceum* is a Gram-negative bacterium commonly used as a reporter for QS activation, since its QS induces the production of violacein, a violet pigment (McClean et al., 1997). The use of two strains, a wild-type strain (ATCC 31532), and an AHL-deficient mutant CV026 strain, allows us to distinguish between QSIs and quenchers of the AHL signal, so-called quorum-quenchers (QQ) (Skogman et al., 2016). In addition to violacein production, we also measured the viability of bacteria, to assess if the inhibition of the QS signal resulted from a genuine QS-inhibitory effect or a bactericidal activity. Etrasimod showed no bactericidal activity against either strain of *C. violaceum*, with less than 10% inhibition of wild-type strain (Figure 5A) and no inhibition of mutant strain, even at the highest concentration tested (Figure 5B). However, etrasimod showed a clear concentration-dependent inhibition of violacein production against both strains. Since it inhibited QS even in the mutant strain (over 70% inhibition at 100 μM), in which external AHL was added to produce violacein, we can conclude that the inhibition occurred downstream of the AHL synthesis, making etrasimod either a mediator of the AHL receptor or a QQ. As the AHL system used in *C. violaceum* is also conserved among many Gram-negative bacteria, this suggests that etrasimod could potentially be used in combination with a bactericidal compound against certain Gram-negative species to prevent the bacteria from forming a biofilm and allowing the second compound to kill the planktonic cells.

### Cytotoxicity

Finally, we evaluated the *in vitro* cytotoxicity of etrasimod by measuring the viability of two mammalian cell lines, A549 and HL-60, after exposure to different concentrations of etrasimod (1–100 μM). Etrasimod showed no toxicity against the two cell lines since no drop of the cell viability was detected even at the highest concentration (100 μM). Meanwhile, the positive control (usnic acid; 100 μM) reduced the viability of HL-60 and A549 cells up to 68.75 ± 5.76 and 70.46 ± 3.57%, respectively. Therefore, these data suggest that etrasimod could be repurposed as an antibiotic, as no cytotoxicity was observed at its MIC against all Gram-positive species tested.

Moreover, the safety profile of etrasimod has been previously studied in both preclinical and clinical settings. In clinical trials, it showed favorable long-term safety for the treatment of ulcerative colitis at a daily dose of 2 mg (Sandborn et al., 2020; Vermeire et al., 2021). No less important, etrasimod has also shown to be well tolerated in rats at doses up to 300 mg/kg, which corresponded to a plasma concentration of 135.7 μM (Buzard et al., 2014). Such studies indicate that etrasimod might be safe in humans even at higher doses than those tested in the recent clinical trials.

## Conclusion

In this study, we screened thirteen commercially available S1PR modulators for their antibacterial activity against *S. aureus* and identified etrasimod as a hit compound with an MIC of 5–10 μM (2.3–4.6 μg/mL). Etrasimod is a drug candidate developed by Arena Pharmaceuticals, Inc. for the treatment of immune-mediated and inflammatory diseases. Further *in vitro* characterization revealed bactericidal activity and a quick decrease in the number of viable bacteria within 1 h of exposure, suggesting that etrasimod affects the bacterial cell by possibly interfering the cell wall synthesis or causing a membrane disruption. This S1PR modulator was also shown to prevent the biofilm formation at its MIC, and it effectively reduced biofilm viability of preformed *S. aureus* biofilms by more than 90% at 100 μM. Etrasimod induced bacterial resistance after 10 days of treatment, suggesting that it could be possibly used for a short-course monotherapy treatment. However, to reduce the risk of resistance development, etrasimod could potentially be used in an antibiotic combination therapy, especially with gentamicin, since this drug combination has shown synergistic activity against *S. aureus*. Furthermore, etrasimod showed strong inhibitory activity with MIC of 5 or 10 μM against a panel of Gram-positive bacteria, including clinical methicillin- and penicillin-resistant *S. aureus* strains, *S. epidermidis*, and *E. faecalis*. On the other hand, while no antibacterial activity was observed against Gram-negative bacteria *A. baumannii*, *P. aeruginosa*, and *E. coli*, it inhibited QS in *C. violaceum*, implying the ability of this compound to block QS in certain Gram-negative bacteria. Finally, etrasimod showed favorable toxicity profile, since no toxicity against mammalian cells was observed up to a concentration of 100 μM, offering a potential therapeutic window for antibacterial treatment.

To the best of our knowledge, this is the first evaluation of etrasimod as a potential antibacterial and anti-biofilm compound and the first report of antibacterial activity of etrasimod against Gram-positive bacteria.

## Data Availability Statement

The original contributions presented in the study are included in the article/Supplementary Material, further inquiries can be directed to the corresponding author.

## Author Contributions

MZ, SG-G, PS-M-G, AF, JY-K, and JZP contributed to conceptualization. MZ, SG-G, and PS-M-G contributed to methodology, investigation, and data analysis. IR caried out cytotoxicity study. MZ wrote the first draft of the manuscript. SG-G, PS-M-G, MZ, KS, LH, AF, JY-K, and JZP contributed to review and editing the manuscript. JZP, AF, KS, and JY-K supervised and revised the work. All authors accepted the final version of the manuscript.

## Conflict of Interest

The authors declare that the research was conducted in the absence of any commercial or financial relationships that could be construed as a potential conflict of interest.

## Publisher’s Note

All claims expressed in this article are solely those of the authors and do not necessarily represent those of their affiliated organizations, or those of the publisher, the editors and the reviewers. Any product that may be evaluated in this article, or claim that may be made by its manufacturer, is not guaranteed or endorsed by the publisher.
