# Supplementary material for: Repurposing the Sphingosine-1-Phosphate Receptor Modulator Etrasimod as an Antibacterial Agent Against Gram-Positive Bacteria
Source: Front Microbiol. 2022 Jun 6;13:926170. doi: 10.3389/fmicb.2022.926170 (PMC9207386; doi:10.3389/fmicb.2022.926170)
Supplement: Supplementary file 1 [file Data_Sheet_1.docx]

Supplementary Material

Repurposing the Sphingosine-1-Phosphate Receptor Modulator Etrasimod as an Antibacterial Agent Against Gram-Positive Bacteria

Matej Zore^1^, Shella Gilbert-Girard^2^, Paola San-Martin-Galindo^2^, Inés Reigada^2^, Leena Hanski^2^, Kirsi Savijoki^2^, Adyary Fallarero^2^, Jari Yli-Kauhaluoma^1^, Jayendra Z. Patel^1*^

^1^Drug Research Program, Division of Pharmaceutical Chemistry and Technology, Faculty of Pharmacy, University of Helsinki, Helsinki, Finland

^2^Drug Research Program, Division of Pharmaceutical Biosciences, Faculty of Pharmacy, University of Helsinki, Helsinki, Finland

*** Correspondence:**Jayendra Z. Patel: jayendra.patel@helsinki.fi

# Supplementary tables

**Table S1:** Initial screening of the sphingosine-1-phosphate receptor (S1PR) modulators at 50 µM against planktonic cells and biofilms of *Staphylococcus aureus* ATCC 25923. Results are expressed as the average inhibition percentage ± SD of two biological repetitions, each with three technical replicates. Fingolimod was used as a reference compound.

| **Compound** | **Structure** | **Average inhibition %** | | | |
| --- | --- | --- | --- | --- | --- |
|  |  | Pre-exposure | | | |
|  |  | Planktonic cells | | Biofilms | |
|  |  | *Turbidity* | *Viability* | *Viability* | *Biomass* |
| 1% DMSO | - | 21.3 ± 21.3 | 4.5 ± 4.5 | 7.7 ± 1.5 | 3.2 ± 2.7 |
| Fingolimod |  | 99.8 ± 0.2 | 99.7 ± 0.1 | 99.8 ± 0.1 | 95.0 ± 0.6 |
| Amiselimod |  | 99.1 ± 0.1 | 99.4 ± 0.0 | 99.7 ± 0.0 | 91.5 ± 0.6 |
| Ceralifimod |  | 18.4 ± 0.6 | 4.4 ± 3.8 | 47.4 ± 0.5 | 31.6 ± 3.1 |
| Etrasimod |  | 99.0 ± 0.0 | 99.6 ± 0.1 | 100.1 ± 0.0 | 95.1 ± 0.3 |
| Cenerimod |  | 43.8 ± 3.6 | 8.9 ± 7.2 | 35.9 ± 1.5 | 19.4 ± 12.7 |
| Siponimod |  | 57.8 ± 5.3 | 42.9 ± 0.3 | 35.1 ± 9.6 | 30.0 ± 7.2 |
| Ozanimod |  | 33.3 ± 5.7 | 14.0 ± 3.7 | 32.3 ± 1.7 | 16.5 ± 2.0 |
| Ponesimod |  | 45.5 ± 6.3 | 15.3 ± 2.7 | 33.7 ± 9.0 | 34.1 ± 5.9 |
| KRP-203 |  | 100.1 ± 0.5 | 99.9 ± 0.2 | 99.7 ± 0.1 | 94.9 ± 0.2 |
| AKP-11 |  | 41.2 ± 11.7 | 24.9 ± 1.4 | 36.5 ± 4.9 | 8.3 ± 5.3 |
| CAY10734 |  | 38.8 ± 14.4 | 12.6 ± 7.6 | 41.2 ± 14.4 | 10.7 ± 10.1 |
| AUY954 |  | 98.6 ± 1.0 | 98.8 ± 0.9 | 93.7 ± 5.1 | 90.3 ± 3.8 |
| GSK2018682 |  | 98.8 ± 1.2 | 98.7 ± 0.8 | 94.3 ± 5.7 | 90.8 ± 4.4 |
| SEW2871 |  | 43.4 ± 30.3 | 27.0 ± 8.2 | 11.0 ± 11.0 | 0.4 ± 0.4 |

**Table S2:** Inhibition of *Staphylococcus aureus* ATCC 25923 in pre-exposure assay by the active S1PR modulators. Results are expressed as the average inhibition percentage ± SD of three biological repetitions, each with two technical replicates per concentration.

| **Compound** | | **Average inhibition %** | | | |
| --- | --- | --- | --- | --- | --- |
|  |  | Pre-exposure | | | |
|  |  | Planktonic cells | | Biofilms | |
|  |  | *Turbidity* | *Viability* | *Viability* | *Biomass* |
| 1% DMSO | | 21.6 ± 4.7 | 15.7 ± 13.3 | 7.8 ± 1.7 | 1.4 ± 1.1 |
| Fingolimod | 2.5 µM | 33.9 ± 24.5 | 17.7 ± 12.6 | 21.4 ± 12.2 | 13.1 ± 4.8 |
|  | 5 µM | 44.0 ± 16.6 | 19.1 ± 14.7 | 26.4 ± 8.5 | 18.2 ± 7.9 |
|  | 10 µM | 48.7 ± 40.9 | 42.5 ± 40.6 | 60.4 ± 29.6 | 51.1 ± 31.6 |
|  | 15 µM | 99.7 ± 0.3 | 99.2 ± 0.5 | 99.9 ± 0.1 | 95.2 ± 1.4 |
|  | 25 µM | 99.9 ± 0.1 | 99.3 ± 0.6 | 98.4 ± 2.0 | 93.1 ± 2.0 |
| Amiselimod | 2.5 µM | 31.1 ± 27.2 | 31.2 ± 9.6 | 23.1 ± 14.5 | 13.4 ± 7.9 |
|  | 5 µM | 44.4 ± 20.5 | 32.6 ± 14.3 | 30.3 ± 18.2 | 23.5 ± 10.8 |
|  | 10 µM | 62.0 ± 26.8 | 47.1 ± 37.3 | 55.5 ± 34.2 | 45.7 ± 37.2 |
|  | 15 µM | 99.5 ± 0.4 | 99.1 ± 0.6 | 99.9 ± 0.2 | 95.7 ± 0.7 |
|  | 25 µM | 99.3 ± 0.3 | 99.0 ± 0.6 | 99.9 ± 0.1 | 94.5 ± 0.7 |
| Etrasimod | 2.5 µM | 26.8 ± 16.1 | 11.7 ± 6.0 | 0.8 ± 1.1 | 0.0 ± 0.0 |
|  | 5 µM | 63.0 ± 28.9 | 51.9 ± 33.0 | 33.8 ± 46.6 | 32.8 ± 46.4 |
|  | 10 µM | 100.1 ± 0.7 | 99.2 ± 0.6 | 100.0 ± 0.1 | 98.2 ± 0.4 |
|  | 15 µM | 99.7 ± 0.6 | 99.2 ± 0.6 | 99.9 ± 0.3 | 97.3 ± 0.3 |
|  | 25 µM | 99.6 ± 0.4 | 99.1 ± 0.5 | 100.2 ± 0.1 | 97.2 ± 0.4 |
| KRP-203 | 2.5 µM | 30.8 ± 22.8 | 25.2 ± 14.3 | 26.8 ± 19.8 | 22.8 ± 19.6 |
|  | 5 µM | 40.4 ± 19.2 | 21.6 ± 13.9 | 33.0 ± 14.0 | 25.2 ± 21.9 |
|  | 10 µM | 36.5 ± 17.3 | 21.1 ± 6.9 | 37.8 ± 10.9 | 21.2 ± 17.8 |
|  | 15 µM | 37.4 ± 13.0 | 13.6 ± 6.2 | 41.4 ± 18.1 | 30.1 ± 21.1 |
|  | 25 µM | 99.7 ± 0.4 | 99.4 ± 0.6 | 100.0 ± 0.2 | 95.9 ± 0.7 |
| AUY954 | 2.5 µM | 24.1 ± 27.6 | 26.8 ± 12.5 | 21.7 ± 13.4 | 6.0 ± 3.5 |
|  | 5 µM | 36.0 ± 30.1 | 32.9 ± 18.9 | 24.1 ± 4.2 | 4.2 ± 5.9 |
|  | 10 µM | 33.4 ± 27.7 | 27.6 ± 18.5 | 32.5 ± 5.7 | 12.8 ± 6.6 |
|  | 15 µM | 33.9 ± 19.7 | 19.7 ± 13.4 | 25.5 ± 5.2 | 8.6 ± 2.2 |
|  | 25 µM | 58.8 ± 14.9 | 37.0 ± 11.3 | 21.3 ± 4.2 | 15.9 ± 11.5 |
| GSK2018682 | 2.5 µM | 10.7 ± 15.2 | 12.9 ± 9.4 | 8.0 ± 7.7 | 7.3 ± 7.0 |
|  | 5 µM | 7.6 ± 5.5 | 5.9 ± 3.8 | 18.6 ± 1.5 | 6.6 ± 9.3 |
|  | 10 µM | 2.7 ± 3.4 | 4.0 ± 3.6 | 25.1 ± 3.9 | 7.4 ± 9.7 |
|  | 15 µM | 98.3 ± 0.1 | 98.8 ± 0.9 | 97.6 ± 1.7 | 92.7 ± 3.6 |
|  | 25 µM | 99.8 ± 0.5 | 99.1 ± 0.8 | 100.0 ± 0.3 | 97.6 ± 0.3 |

**Table S3:** Inhibition of *Staphylococcus aureus* ATCC 25923 in post-exposure assay by the active S1PR modulators. Results are expressed as the average inhibition percentage ± SD of two biological repetitions, each with three technical replicates per concentration.

| **Compound** | | **Average inhibition %** | | | |
| --- | --- | --- | --- | --- | --- |
|  |  | Post-exposure | | | |
|  |  | Planktonic cells | | Biofilms | |
|  |  | *Turbidity* | *Viability* | *Viability* | *Biomass* |
| 1% DMSO | | 8.9 ± 0.1 | 0.5 ± 0.5 | 0.2 ± 0.2 | 2.8 ± 2.8 |
| Fingolimod | 25 µM | 33.4 ± 14.8 | 12.7 ± 11.1 | 15.2 ± 3.3 | 31.8 ± 13.6 |
|  | 50 µM | 55.8 ± 7.5 | 33.9 ± 22.4 | 43.0 ± 4.4 | 41.1 ± 9.6 |
|  | 100 µM | 79.1 ± 10.8 | 77.9 ± 21.3 | 52.6 ± 21.2 | 42.9 ± 13.5 |
|  | 200 µM | 86.7 ± 6.5 | 94.9 ± 4.8 | 79.2 ± 19.6 | 55.6 ± 14.6 |
| Amiselimod | 25 µM | 37.5 ± 18.8 | 21.3 ± 21.3 | 1.3 ± 1.3 | 13.4 ± 9.1 |
|  | 50 µM | 64.5 ± 1.9 | 42.7 ± 14.6 | 20.3 ± 1.7 | 13.9 ± 2.3 |
|  | 100 µM | 84.9 ± 3.7 | 99.4 ± 0.2 | 73.7 ± 24.6 | 53.9 ± 11.0 |
|  | 200 µM | 77.9 ± 5.9 | 99.3 ± 0.0 | 99.9 ± 0.6 | 69.5 ± 1.2 |
| Etrasimod | 25 µM | 13.6 ± 12.3 | 11.6 ± 11.6 | 3.6 ± 1.3 | 6.4 ± 0.7 |
|  | 50 µM | 39.6 ± 31.7 | 28.0 ± 28.0 | 19.2 ± 14.6 | 16.4 ± 3.7 |
|  | 100 µM | 90.0 ± 6.3 | 97.9 ± 1.6 | 94.3 ± 5.6 | 13.1 ± 10.4 |
|  | 200 µM | 89.9 ± 4.7 | 99.7 ± 0.1 | 99.0 ± 1.5 | 3.9 ± 0.3 |
| KRP-203 | 25 µM | 35.9 ± 9.3 | 11.7 ± 5.5 | 8.4 ± 4.2 | 26.3 ± 5.8 |
|  | 50 µM | 68.4 ± 9.1 | 77.1 ± 5.0 | 9.3 ± 2.7 | 38.9 ± 1.6 |
|  | 100 µM | 84.0 ± 3.0 | 99.7 ± 0.1 | 84.2 ± 14.5 | 63.4 ± 1.7 |
|  | 200 µM | 87.1 ± 1.1 | 99.9 ± 0.1 | 92.8 ± 7.2 | 57.4 ± 2.8 |
| AUY954 | 25 µM | 32.0 ± 3.9 | 11.9 ± 5.2 | 3.0 ± 1.4 | 13.3 ± 5.1 |
|  | 50 µM | 41.7 ± 4.5 | 27.8 ± 11.6 | 6.7 ± 1.5 | 15.7 ± 0.9 |
|  | 100 µM | 55.7 ± 8.8 | 38.3 ± 3.1 | 1.3 ± 0.1 | 17.8 ± 3.3 |
|  | 200 µM | 55.3 ± 14.0 | 50.6 ± 10.2 | 0.0 ± 0.0 | 17.4 ± 1.6 |
| GSK2018682 | 25 µM | 17.2 ± 5.2 | 11.2 ± 0.7 | 17.5 ± 6.4 | 19.4 ± 6.1 |
|  | 50 µM | 10.1 ± 10.1 | 10.2 ± 8.7 | 12.2 ± 7.6 | 25.0 ± 2.7 |
|  | 100 µM | 91.4 ± 1.6 | 98.9 ± 0.4 | 94.6 ± 0.2 | 19.2 ± 10.7 |
|  | 200 µM | 63.8 ± 10.5 | 96.0 ± 3.4 | 88.7 ± 11.0 | 10.9 ± 3.0 |

**Table S4:** Inhibition of Gram-positive bacteria in pre-exposure assay by etrasimod. Results are expressed as the average inhibition percentage ± SD of three biological repetitions, each with two technical replicates per concentration.

|  | | **Average inhibition %** | | | |
| --- | --- | --- | --- | --- | --- |
|  |  | ***Staphylococcus aureus* Newman** | | | |
|  |  | Planktonic cells | | Biofilms | |
|  |  | *Turbidity* | *Viability* | *Viability* | *Biomass* |
| 1% DMSO | | 26.3 ± 10.4 | 8.0 ± 10.3 | 8.7 ± 1.8 | 6.1 ± 8.4 |
| Etrasimod | 2.5 µM | 3.4 ± 4.9 | 15.2 ± 21.1 | 1.2 ± 1.4 | 0.0 ± 0.0 |
|  | 5 µM | 99.4 ± 0.4 | 99.4 ± 0.3 | 97.8 ± 2.8 | 93.0 ± 5.8 |
|  | 10 µM | 99.7 ± 1.1 | 99.7 ± 0.1 | 99.9 ± 0.0 | 97.0 ± 0.4 |
|  | 15 µM | 99.2 ± 0.7 | 99.5 ± 0.0 | 99.9 ± 0.0 | 96.7 ± 0.6 |
|  | 25 µM | 98.6 ± 0.8 | 99.4 ± 0.1 | 99.9 ± 0.1 | 96.6 ± 0.7 |
|  | 50 µM | 96.6 ± 0.3 | 99.2 ± 0.1 | 99.8 ± 0.1 | 96.2 ± 0.5 |
|  | | ***Staphylococcus aureus* P2** | | | |
|  |  | Planktonic cells | | Biofilms | |
|  |  | *Turbidity* | *Viability* | *Viability* | *Biomass* |
| 1% DMSO | | 35.8 ± 1.4 | 1.4 ± 2.0 | 0.0 ± 0.0 | 0.0 ± 0.0 |
| Etrasimod | 2.5 µM | 9.8 ± 1.8 | 19.2 ± 23.5 | 4.8 ± 3.4 | 12.6 ± 3.6 |
|  | 5 µM | 99.7 ± 0.1 | 99.5 ± 0.2 | 98.2 ± 2.2 | 95.4 ± 3.7 |
|  | 10 µM | 99.5 ± 0.2 | 99.7 ± 0.0 | 99.8 ± 0.1 | 97.4 ± 0.2 |
|  | 15 µM | 99.3 ± 0.3 | 99.5 ± 0.1 | 99.8 ± 0.1 | 97.8 ± 1.0 |
|  | 25 µM | 98.8 ± 0.2 | 99.5 ± 0.0 | 99.7 ± 0.2 | 97.2 ± 0.4 |
|  | 50 µM | 97.6 ± 0.8 | 99.5 ± 0.1 | 99.7 ± 0.1 | 96.0 ± 0.4 |
|  | | ***Staphylococcus aureus* ATCC 43300 (MRSA)** | | | |
|  |  | Planktonic cells | | Biofilms | |
|  |  | *Turbidity* | *Viability* | *Viability* | *Biomass* |
| 1% DMSO | | 26.4 ± 13.0 | 0.0 ± 0.0 | 0.0 ± 0.0 | 4.6 ± 3.7 |
| Etrasimod | 2.5 µM | 14.4 ± 10.7 | 9.7 ± 7.3 | 0.2 ± 0.3 | 4.3 ± 6.0 |
|  | 5 µM | 99.6 ± 0.2 | 99.6 ± 0.1 | 96.5 ± 4.5 | 94.6 ± 3.1 |
|  | 10 µM | 99.5 ± 0.3 | 99.6 ± 0.2 | 99.9 ± 0.1 | 97.3 ± 0.5 |
|  | 15 µM | 99.3 ± 0.2 | 99.5 ± 0.1 | 99.9 ± 0.1 | 96.2 ± 0.7 |
|  | 25 µM | 98.6 ± 0.6 | 99.5 ± 0.1 | 99.8 ± 0.2 | 95.8 ± 0.4 |
|  | 50 µM | 97.3 ± 0.5 | 99.5 ± 0.1 | 99.7 ± 0.2 | 94.7 ± 0.4 |
|  | | ***Staphylococcus epidermidis* RP62A** | | | |
|  |  | Planktonic cells | | Biofilms | |
|  |  | *Turbidity* | *Viability* | *Viability* | *Biomass* |
| 1% DMSO | | 45.1 ± 5.7 | 39.2 ± 11.6 | 0.3 ± 0.5 | 0.0 ± 0.0 |
| Etrasimod | 2.5 µM | 17.4 ± 4.6 | 17.7 ± 6.6 | 3.0 ± 4.2 | 1.6 ± 2.0 |
|  | 5 µM | 9.8 ± 13.9 | 6.7 ± 8.0 | 23.7 ± 9.7 | 23.5 ± 10.8 |
|  | 10 µM | 99.2 ± 0.4 | 99.5 ± 0.2 | 99.9 ± 0.1 | 99.7 ± 0.2 |
|  | 15 µM | 98.2 ± 0.4 | 99.4 ± 0.1 | 99.8 ± 0.2 | 99.1 ± 0.1 |
|  | 25 µM | 98.3 ± 0.3 | 99.3 ± 0.2 | 99.9 ± 0.1 | 99.0 ± 0.0 |
|  | 50 µM | 95.5 ± 1.8 | 99.4 ± 0.2 | 99.8 ± 0.1 | 98.5 ± 0.1 |
|  | | ***Staphylococcus epidermidis* P55** | | | |
|  |  | Planktonic cells | | Biofilms | |
|  |  | *Turbidity* | *Viability* | *Viability* | *Biomass* |
| 1% DMSO | | 1.5 ± 2.1 | 3.3 ± 2.3 | 0.0 ± 0.0 | 5.1 ± 3.7 |
| Etrasimod | 2.5 µM | 7.0 ± 5.3 | 8.8 ± 4.4 | 0.0 ± 0.0 | 18.2 ± 8.5 |
|  | 5 µM | 81.2 ± 1.7 | 78.0 ± 18.3 | 40.7 ± 25.3 | 38.2 ± 14.8 |
|  | 10 µM | 99.5 ± 0.3 | 99.7 ± 0.1 | 99.9 ± 0.2 | 99.9 ± 0.1 |
|  | 15 µM | 99.2 ± 0.3 | 99.6 ± 0.1 | 92.3 ± 10.6 | 85.1 ± 19.2 |
|  | 25 µM | 99.1 ± 0.1 | 99.5 ± 0.1 | 100.1 ± 0.1 | 97.8 ± 0.2 |
|  | 50 µM | 98.3 ± 0.4 | 99.6 ± 0.1 | 100.1 ± 0.3 | 96.1 ± 0.8 |
|  | | ***Enterococcus faecalis* ATCC 29212** | | | |
|  |  | Planktonic cells | | Biofilms | |
|  |  | *Turbidity* | *Viability* | *Viability* | *Biomass* |
| 1% DMSO | | 14.0 ± 14.2 | 0.0 ± 0.0 | 2.9 ± 3.2 | 12.6 ± 4.2 |
| Etrasimod | 2.5 µM | 46.5 ± 5.0 | 3.1 ± 4.4 | 0.0 ± 0.0 | 16.4 ± 11.3 |
|  | 5 µM | 100.4 ± 0.9 | 91.3 ± 0.5 | 96.7 ± 2.2 | 99.1 ± 0.1 |
|  | 10 µM | 100.5 ± 1.1 | 102.6 ± 1.0 | 99.2 ± 0.7 | 99.5 ± 0.1 |
|  | 15 µM | 100.3 ± 1.1 | 102.8 ± 1.6 | 100.3 ± 0.3 | 99.4 ± 0.2 |
|  | 25 µM | 98.9 ± 0.9 | 102.3 ± 1.9 | 100.8 ± 0.8 | 99.2 ± 0.2 |
|  | 50 µM | 98.4 ± 0.2 | 101.8 ± 3.2 | 100.2 ± 0.2 | 99.8 ± 0.4 |

**Table S5:** Inhibition of Gram-negative bacteria in pre-exposure assay by etrasimod. Results are expressed as the average inhibition percentage ± SD of two biological repetitions, each with three technical replicates per concentration.

|  | | **Average inhibition %** | | | |
| --- | --- | --- | --- | --- | --- |
|  |  | ***Acinetobacter baumannii* NCTC 13423** | | | |
|  |  | Planktonic cells | | Biofilms | |
|  |  | *Turbidity* | *Viability* | *Viability* | *Biomass* |
| 1% DMSO | | 10.9 ± 0.4 | 3.8 ± 3.8 | 1.3 ± 1.3 | 0.0 ± 0.0 |
| Etrasimod | 10 µM | 2.8 ± 2.8 | 1.3 ± 1.3 | 0.5 ± 0.5 | 8.6 ± 8.6 |
|  | 15 µM | 5.2 ± 5.2 | 4.1 ± 4.1 | 0.0 ± 0.0 | 2.3 ± 1.9 |
|  | 25 µM | 3.6 ± 3.6 | 5.5 ± 4.4 | 10.3 ± 2.9 | 3.0 ± 3.0 |
|  | 50 µM | 4.1 ± 4.1 | 5.6 ± 5.3 | 14.8 ± 9.4 | 0.7 ± 0.7 |
|  | 100 µM | 2.2 ± 2.2 | 6.9 ± 6.5 | 12.6 ± 3.8 | 0.0 ± 0.0 |
|  | 200 µM | 0.3 ± 0.3 | 12.6 ± 12.3 | 0.0 ± 0.0 | 0.0 ± 0.0 |
|  | | ***Escherichia coli* ATCC BAA1161** | | | |
|  |  | Planktonic cells | | Biofilms | |
|  |  | *Turbidity* | *Viability* | *Viability* | *Biomass* |
| 1% DMSO | | 0.0 ± 0.0 | 0.0 ± 0.0 | 5.7 ± 5.7 | 12.9 ± 10.3 |
| Etrasimod | 10 µM | 0.0 ± 0.0 | 0.0 ± 0.0 | 15.9 ± 15.9 | 42.7 ± 0.4 |
|  | 15 µM | 1.5 ± 1.5 | 0.0 ± 0.0 | 20.1 ± 20.1 | 44.1 ± 13.4 |
|  | 25 µM | 3.9 ± 0.2 | 0.0 ± 0.0 | 20.9 ± 20.3 | 50.8 ± 3.9 |
|  | 50 µM | 10.5 ± 1.7 | 2.2 ± 2.2 | 18.4 ± 18.4 | 44.7 ± 8.3 |
|  | 100 µM | 12.6 ± 7.9 | 6.6 ± 6.6 | 4.3 ± 4.3 | 37.5 ± 8.3 |
|  | 200 µM | 17.2 ± 17.2 | 13.9 ± 13.9 | 0.0 ± 0.0 | 26.4 ± 20.8 |
|  | | ***Pseudomonas aeruginosa* ATCC 15442** | | | |
|  |  | Planktonic cells | | Biofilms | |
|  |  | *Turbidity* | *Viability* | *Viability* | *Biomass* |
| 1% DMSO | | 7.1 ± 1.5 | 15.9 ± 2.1 | 0.0 ± 0.0 | 0.0 ± 0.0 |
| Etrasimod | 10 µM | 2.4 ± 2.4 | 1.1 ± 1.1 | 3.7 ± 1.2 | 9.4 ± 6.3 |
|  | 15 µM | 3.0 ± 3.0 | 2.4 ± 2.4 | 3.7 ± 3.7 | 6.6 ± 4.3 |
|  | 25 µM | 3.4 ± 3.2 | 2.5 ± 1.5 | 2.5 ± 2.5 | 8.4 ± 6.6 |
|  | 50 µM | 6.8 ± 4.7 | 2.8 ± 0.2 | 2.7 ± 2.7 | 10.3 ± 4.9 |
|  | 100 µM | 15.6 ± 2.3 | 0.0 ± 0.0 | 0.0 ± 0.0 | 1.6 ± 1.6 |
|  | 200 µM | 31.9 ± 0.9 | 4.0 ± 2.5 | 1.1 ± 1.1 | 0.2 ± 0.2 |
|  | | ***Pseudomonas aeruginosa* PAO1** | | | |
|  |  | Planktonic cells | | Biofilms | |
|  |  | *Turbidity* | *Viability* | *Viability* | *Biomass* |
| 1% DMSO | | 12.5 ± 0.1 | 4.5 ± 4.5 | 0.0 ± 0.0 | 9.3 ± 3.1 |
| Etrasimod | 10 µM | 11.4 ± 2.1 | 3.7 ± 1.5 | 8.8 ± 3.4 | 9.4 ± 5.4 |
|  | 15 µM | 14.2 ± 3.0 | 17.4 ± 7.9 | 13.4 ± 11.4 | 10.5 ± 2.4 |
|  | 25 µM | 11.8 ± 0.4 | 12.3 ± 3.3 | 14.0 ± 14.0 | 7.4 ± 2.1 |
|  | 50 µM | 0.0 ± 0.0 | 16.1 ± 6.1 | 9.7 ± 9.7 | 7.1 ± 4.2 |
|  | 100 µM | 0.0 ± 0.0 | 0.0 ± 0.0 | 13.5 ± 13.5 | 5.4 ± 4.0 |
|  | 200 µM | 0.0 ± 0.0 | 0.0 ± 0.0 | 25.3 ± 13.4 | 36.5 ± 3.2 |
